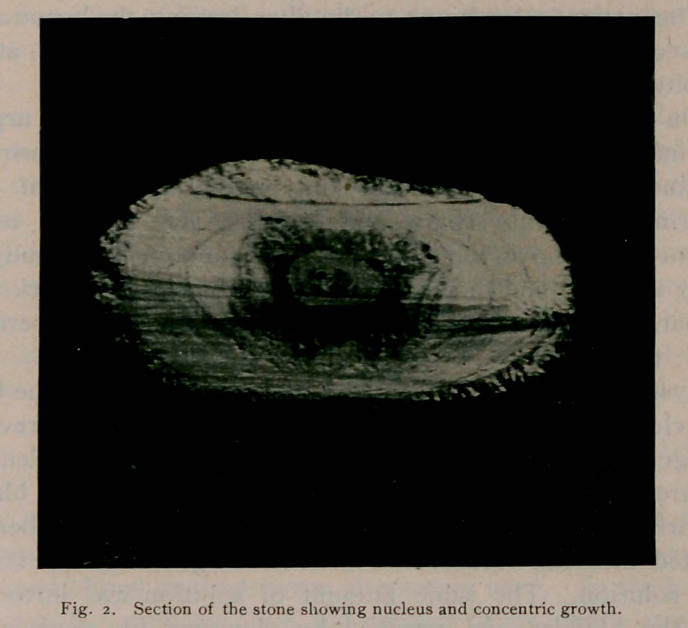# Genitourinary Cases

**Published:** 1902-07

**Authors:** J. A. Gardner, Nelson W. Wilson

**Affiliations:** Buffalo, N. Y.; Genitourinary surgeons to the Emergency Hospital.; Buffalo, N. Y.; Genitourinary surgeons to the Emergency Hospital


### CLINICAL REPORT.

### Genitourinary Cases.

From the services of Dr. J. A. GARDNER and Dr. NELSON W. WILSON, Buffalo, N. Y.
Genitourinary surgeons to the Emergency Hospital.

### LARGE STONE IN THE BLADDER.

THE chain of circumstances leading up to this young man's admission to the genitourinary department of the Emergency Hospital was so unique that it attracted more attention during his recital than his apparent condition. He is 22 years old, single and an upholsterer. He came into the hospital complaining of constant pain in his bladder, a sense of soreness through his penis, and a constant desire to urinate which was unrelieved by the few drops of urine which were expelled from time to time. He was pale, emaciated and shaky, his eyes sunken and his lips cracked and dry. His appearance was typically that of a tubercular patient en route to the grave. His discouragement was deep, he looked upon himself as hopelessly lost to health and was possessed of a deep-rooted fear of impending death. The first questions regarding his family history revealed the hopelessness of his own views of his case.

"Ain't no use asking me nothin' about my folks. They none of them never had anything like this. They are all healthy. I'm the only one that's no good, and I been trying to get better for five years and I've had more than ten doctors fixing me up all the time. It ain't no use. All I want fixed now is my bladder so I can make water without wantin' to and not bein' able to all the time."

Persistent questioning brought out the facts that his father had died at 40 of pneumonia, his mother at 47 of fatty "regeneration" of the heart; three brothers and two sisters are living and all are well. Five years ago the patient first noticed genitourinary trouble. There was considerable scrotal itching and frequency of urination with some pain at the end of the act. He went to a doctor who gave him medicine which relieved him temporarily. With the return of the trouble a few days later he went back to the doctor and was informed that he had gonorrhea and was given pills and an injection. He juggled back and

Fig. 1. The stone. Natural size. (The horizontal line is where the stone was cut through.)

forth with this treatment for the greater part of a year until the pain began to run up his back to his kidneys and the desire to urinate became so frequent that he was kept busy the greater part of the day and night in a vain endeavor to empty his bladder. Then, too, the pain got so bad that he would have to stop walking and rest until the paroxysm passed or eased down. About this time he made up his mind that the doctor did not know what to do for him. This view was corroborated by the "eminent specialist" whose advertisements in the daily newspapers give "comfort to countless thousands," who told him he was suffering with "loss of manhood and stricture of the bladder," and that the only thing that would give him relief was a certain medicine and treatments at so much per month according to a sliding scale. That looked fair to the suffering patient and he took the "road to health and happiness," which ended when he noticed drops of blood at the urinary meatus at the end of micturition. He abruptly quit the man who "cures to stay cured" and transferred his allegiance to the "friend of suffering mankind," whose consultations are free and who charges "a small fee for the medicines."

Here it was discovered that the patient had "weakness of the kidneys and bladder trouble." For some months he poured

Fig. 2. Section of the stone showing nucleus and concentric growth.

into his system the medicines for which *small charges* were made. He took pills and powders and liquids and pretty nearly everything else, according to his description, except hair tonics and corn plasters. As the symptoms which originally disturbed his peace of mind and bodily comfort were aggravated by time and lack of treatment he lost faith in his medical adviser and numbered himself among the hosts of devotees at the shrine of the electric belt, "the gentle current of which passing through the kidneys night and day" would soon effect a cure. Strange to say as soon as he got the belt on all his symptoms disappeared and he felt quite different. This lasted for something like five minutes. The belt lasted about a month and then when a particularly free flow of blood alarmed him he cast it aside and spent the next six months endeavoring to drink up all the kidney cures he could get his hands on. He became an omnivorous reader of the daily newspapers; medical advertisements filled his pockets and he lived in hopes, but got worse with each bottle. He finally landed in a hospital in this city where his trouble was diagnosticated as stricture of the urethra and sounds were sed. It is only fair to state here, that the diagnosis was made by an interne who neglected to refer the case to the attending surgeon at any time during the three days the man was in the hospital ward.

The patient went home to die after leaving the hospital and was sent to the genitourinary department by Dr. Dorr, attending physician at the Emergency, who suspected stone.

On entry the man's symptoms were: frequency and urgency of urination with expulsion of a few drops at a time; desire and tenesmus constant; hematuria and pain at the end of effort at urination. Pain sharp and burning and referred to the rectum and above the pubes. Urine ammoniacal, cloudy and heavy with pus and mucus; after straining at stool a thick white discharge would issue from the urinary meatus. Temperature, 102.1; pulse, 128.

Cystoscopic examination was negative because of the blood and cloudy bladder contents. A Thompson searcher revealed a large stone on the right side of the bladder. The patient was prepared and operation done the next morning. The bladder was irrigated with boracic acid solution and a rubber bag inserted into the rectum and filled with eight ounces of boracic acid solution. The same amount of solution was introduced into the bladder and retained by clamping the penis with a Wilson-Gardner clamp. A vertical incision 4.4 c.m. was made above the pubes, carried between the recti muscles down to the perivesical space. The fat was pushed up with the fingers and the bladder exposed, opened and anchored with silk sutures. On exploration with the finger the stone was found attached to the right side of the bladder and was loosened and removed with forceps with difficulty. Owing to the dirty condition of the bladder, packing of sterile gauze was used and dry dressing applied. This dressing was changed every two hours. The patient made an uninterrupted recovery and at no time after the operation was his temperature over 100°. The stone is mulberry shaped; weighs 112.50 gms., is 6.2 c.m. in length at the widest part and 3.1 in width. The specimen shows beautifully the formation with its nucleus of uric acid and concentric layers of phosphates.

### SOME OF THE EFFECTS OF MASTURBATION.

A case which has been under treatment at the hands of a general practitioner for several months was sent to the hospital for examination and diagnosis. The patient is 19 years old, had frequent urination with dull pain over the pubes at begining which "gradually faded away." Some months ago there had been a trace of blood. Examination shows normal testicles and cord; no pain on pressure anywhere externally. Penis flabby, with long foreskin. Internal examination: tender urethra; bladder, clean; prostate, enlarged and slightly tender; right seminal vesicle distended. There was no history of venereal disease. No sexual intercourse for the past eighteen months. Vesiculitis and subacute prostatitis due to masturbation was the diagnosis. The patient was put on belladonna and opium rectal suppositories with neurosine internally. Later the vesicle was stripped and the prostate massaged and he was circumcised. He was plainly advised of the dangers of autoorgasm. All symptoms have disappeared and he is cured.

### THE BEST METHOD OF INFANT CIRCUMCISION.

In eighteen infantile circumcisions, the eldest child being between 3 and 4 months old and the youngest 18 hours, the open and closed methods were alternately used. In the former there was no suturing done, the prepuce being merely trimmed off back of the glans and the wound being left open. All bleeding points were nipped with hemostatic forceps, the wound dusted with a mixture of orthoform and vitrogen and covered with cotton. The others were sutured after cutting and dressed in the usual way. In every case there was less apparent pain, less discomfort and disturbance, more raipd healing and a better appearing penis where the open method was used. In those cases where the sutures were used the stitches apparently caused a great deal of irritation and restlessness. The peaceful, uninterrupted healing of the wound where sutures were not used makes it the ideal method in very young infants.

A CHEAP AND HANDY APPLICATOR.

A handy and cheap instrument for genitourinary work is the ordinary cotton-tipped pipe cleaner, which may be purchased in any cigar store for 5 cents a dozen. The urethra may be wiped out with these and ointments applied with the utmost ease and safety. They have been in use in the genito-urinary dispensary for some months and have been found serviceable and trustworthy.

49 NIAGARA STREET.

## Figures and Tables

**Fig. I. f1:**
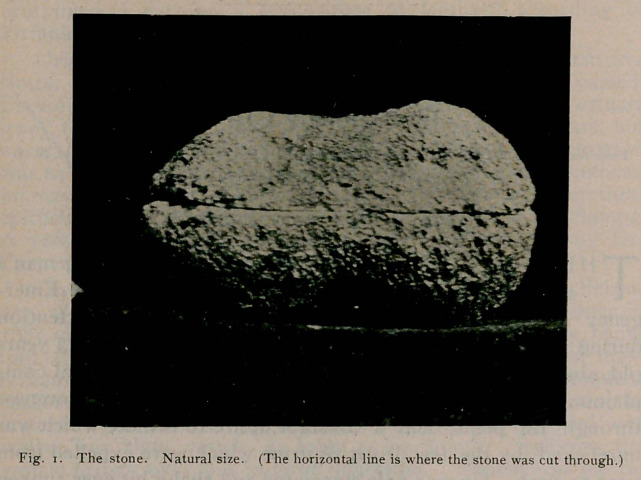


**Fig. 2. f2:**